# Investigating Cardiac Motion Patterns Using Synthetic High-Resolution 3D Cardiovascular Magnetic Resonance Images and Statistical Shape Analysis

**DOI:** 10.3389/fped.2017.00034

**Published:** 2017-03-08

**Authors:** Benedetta Biffi, Jan L. Bruse, Maria A. Zuluaga, Hopewell N. Ntsinjana, Andrew M. Taylor, Silvia Schievano

**Affiliations:** ^1^Centre for Cardiovascular Imaging, UCL Institute of Cardiovascular Science & Great Ormond Street Hospital for Children, London, UK; ^2^Department of Medical Physics and Biomedical Engineering, University College London, London, UK; ^3^Translational Imaging Group, Centre for Medical Image Computing, University College London, London, UK

**Keywords:** ventricular mechanics, congenital heart disease, cardiac magnetic resonance, automatic segmentation, statistical shape analysis

## Abstract

Diagnosis of ventricular dysfunction in congenital heart disease is more and more based on medical imaging, which allows investigation of abnormal cardiac morphology and correlated abnormal function. Although analysis of 2D images represents the clinical standard, novel tools performing automatic processing of 3D images are becoming available, providing more detailed and comprehensive information than simple 2D morphometry. Among these, statistical shape analysis (SSA) allows a consistent and quantitative description of a population of complex shapes, as a way to detect novel biomarkers, ultimately improving diagnosis and pathology understanding. The aim of this study is to describe the implementation of a SSA method for the investigation of 3D left ventricular shape and motion patterns and to test it on a small sample of 4 congenital repaired aortic stenosis patients and 4 age-matched healthy volunteers to demonstrate its potential. The advantage of this method is the capability of analyzing subject-specific motion patterns separately from the individual morphology, visually and quantitatively, as a way to identify functional abnormalities related to both dynamics and shape. Specifically, we combined 3D, high-resolution whole heart data with 2D, temporal information provided by cine cardiovascular magnetic resonance images, and we used an SSA approach to analyze 3D motion *per se*. Preliminary results of this pilot study showed that using this method, some differences in end-diastolic and end-systolic ventricular shapes could be captured, but it was not possible to clearly separate the two cohorts based on shape information alone. However, further analyses on ventricular motion allowed to qualitatively identify differences between the two populations. Moreover, by describing shape and motion with a small number of principal components, this method offers a fully automated process to obtain visually intuitive and numerical information on cardiac shape and motion, which could be, once validated on a larger sample size, easily integrated into the clinical workflow. To conclude, in this preliminary work, we have implemented state-of-the-art automatic segmentation and SSA methods, and we have shown how they could improve our understanding of ventricular kinetics by visually and potentially quantitatively highlighting aspects that are usually not picked up by traditional approaches.

## 1 Introduction

Imaging plays a crucial role in the diagnosis of congenital heart disease (CHD), allowing investigation of complex morphology and correlated pathophysiology (1). In particular, echocardiography and cardiovascular magnetic resonance (CMR) are used to directly or indirectly derive parameters (e.g., ejection fraction, valve inflow profile, strain, and strain rate) used to describe myocardial shape and kinetics, aiding in the diagnosis of ventricular dysfunction (2–5). While advanced image modalities can provide detailed 3D anatomical data, their analysis in clinical practice is often limited to simple 2D morphometry, which does not take into consideration the contribution of the third dimension. Moreover, the estimation of useful parameters from medical images is mostly performed using manual methods, which are time consuming and strongly rely on the specific expertise of the operator, therefore prone to human error. This does not allow for image-processing standardization and prevents the adoption of more complex analyses in routine clinical practice.

Recently, sophisticated engineering techniques for automatic medical image processing have been developed, which provide faster and more accurate ways of extracting the large amount of 3D shape and temporal information carried by medical images. This large amount of data is naturally suited for computer-based analyses, such as statistical shape analysis (SSA) (6), a tool that provides a consistent and quantitative technique of describing complex shapes, ultimately leading to the discovery of novel shape biomarkers or unexpected trends, clusters, or outliers (7). By correlating cardiac shape with clinical or functional parameters by means of regression or classification techniques, SSA can also be used in a predictive way (8–12). To date, most SSA studies have focused on 2D cine images to explore motion features for diagnostic or prognostic purposes (13–16), hence potentially losing crucial 3D information that can only be provided by high-resolution volumetric images, such as whole heart (WH) datasets.

Here, we present a novel method that (i) combines 3D, high-resolution WH data with 2D, temporal information provided by cine images, to extract the most complete information out of the data clinically acquired by CMR and (ii) uses a SSA approach to consistently analyze 3D motion *per se*, allowing to gain insight into ventricular kinetics by visually and quantitatively highlighting aspects of ventricular motion that are usually not picked up by traditional approaches. The wide anatomical and functional variations encountered in CHD are ideal to test the capability of this new tool for independent assessment of shape and motion. In this study, we first present the implementation of an automatic pipeline for the processing of volumetric WH and dynamic cine CMR images, where the motion information provided by the cine is propagated onto the WH images. By exploiting an automatic, atlas-based segmentation method and a shape analysis tool, our pipeline can generate a computational surface mesh of the left ventricle (LV) and parametrize the movement of each subject LV during the cardiac cycle. The principal contributors of ventricular shape and motion, generated from the obtained surface meshes and shape deformations via principal component analysis (PCA), are finally presented with powerful visualization tools. We show the potential of the developed methodology to qualitatively and quantitatively evaluate the difference in both LV 3D shape and motion patterns between healthy subjects and congenital repaired aortic stenosis (AS) patients characterized by LV dysfunction. Results from such analysis may provide novel shape and motion biomarker information, which could ultimately improve the understanding of complex cardiac disease.

## 2 Materials and Methods

### 2.1 Population and Images

Clinically acquired data of 4 congenital repaired aortic stenosis (AS) patients (14 ± 2 years) and 4 age-matched (17 ± 3 years) healthy volunteers (control group) were retrospectively used for this study. Ethical approval was obtained by the Institute of Child Health/Great Ormond Street Hospital for Children Research Ethics Committee, and all patients or legal parent or guardian gave informed consent for research use of the data. The patients were diagnosed with chronic ventricular dysfunction as a sequel of congenital aortic stenosis (2 neonatal surgical valvotomy followed by Ross procedure, 1 neonatal balloon aortic valvuloplasty followed by Ross procedure, 1 neonatal surgical valvotomy followed by balloon aortic valvuloplasty). Age, LV end-diastolic volume (EDV), and ejection fraction (EF) of the sample population are shown in Table 1. CMR data were acquired at Great Ormond Street Hospital for Children (Great Ormond Street, London, UK) with a 1.5-T scanner (Avanto, Siemens Medical Solutions, Erlangen, Germany), in the patients as part of the clinical follow-up. For each subject, the following two sets of images were used in this study: (i) balanced steady-state free precession (bSSFP) 3D WH (isotropic voxel size of 1.4 mm), acquired during the mid-diastolic rest period in free breathing and with ECG- and respiratory-gating and (ii) retrospectively gated bSSFP cine images, acquired in breath-hold (~20 frames per cardiac cycle) in the short-axis (SAX) from the valves plane to the apex (slice spacing ~8 mm, in plane isotropic voxel size of 1.4 mm).

**Table 1:** **Age, LV EDV, and EF of the 4 AS patients and the 4 healthy volunteers analyzed in this study**.

	Age (years)	EDV (mL)	EF (%)
Control_1_	19	151	63
Control_2_	13	130	73
Control_3_	20	119	62
Control_4_	16	96	66
AS_1_	14	113	80
AS_2_	14	147	51
AS_3_	17	132	68
AS_4_	11	64	68

### 2.2 Workflow

**Figure 1:**
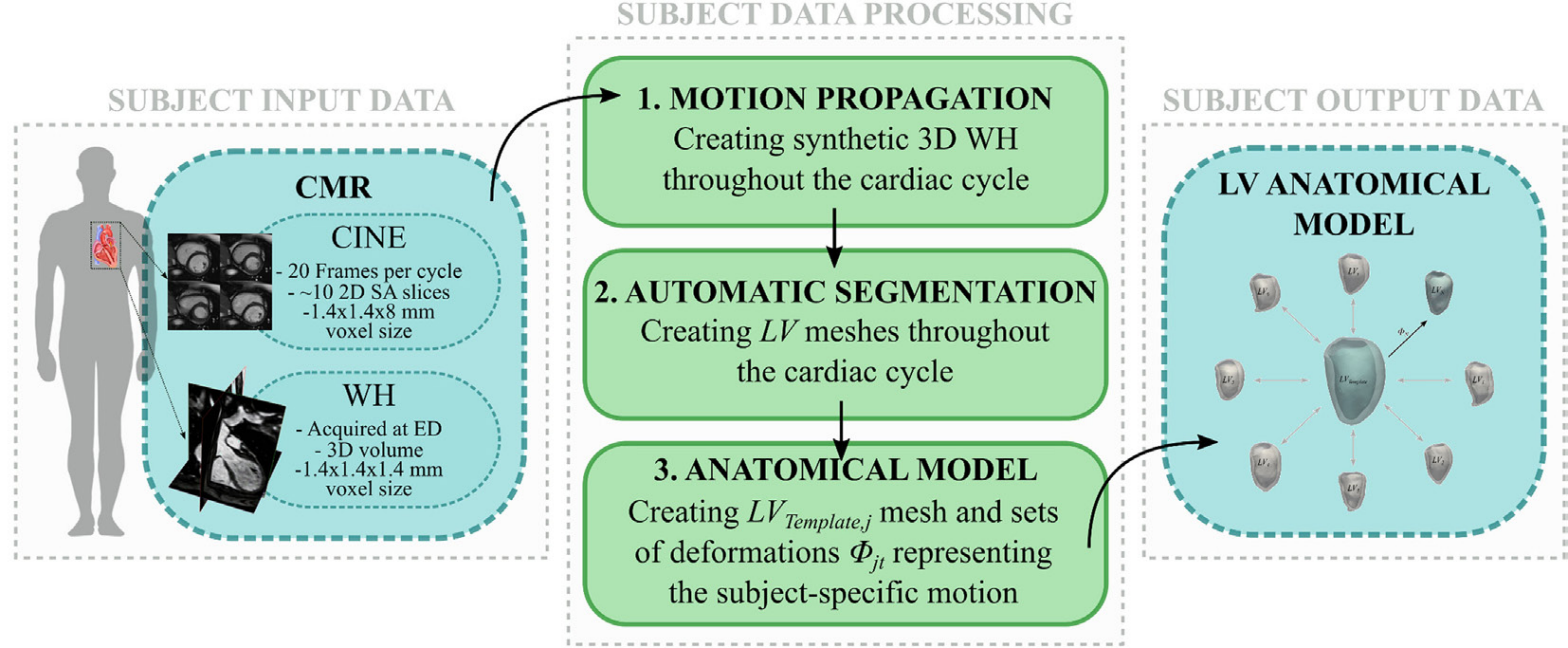
**Image-processing pipeline to generate each subject LV anatomical model starting from cine and WH CMR images: (1) Synthetic WH images were created by combining WH with the cine images (2) and segmented with our automatic segmentation method; (3) LV meshes were exported and used to create the subject LV anatomical model**.

**Figure 2:**
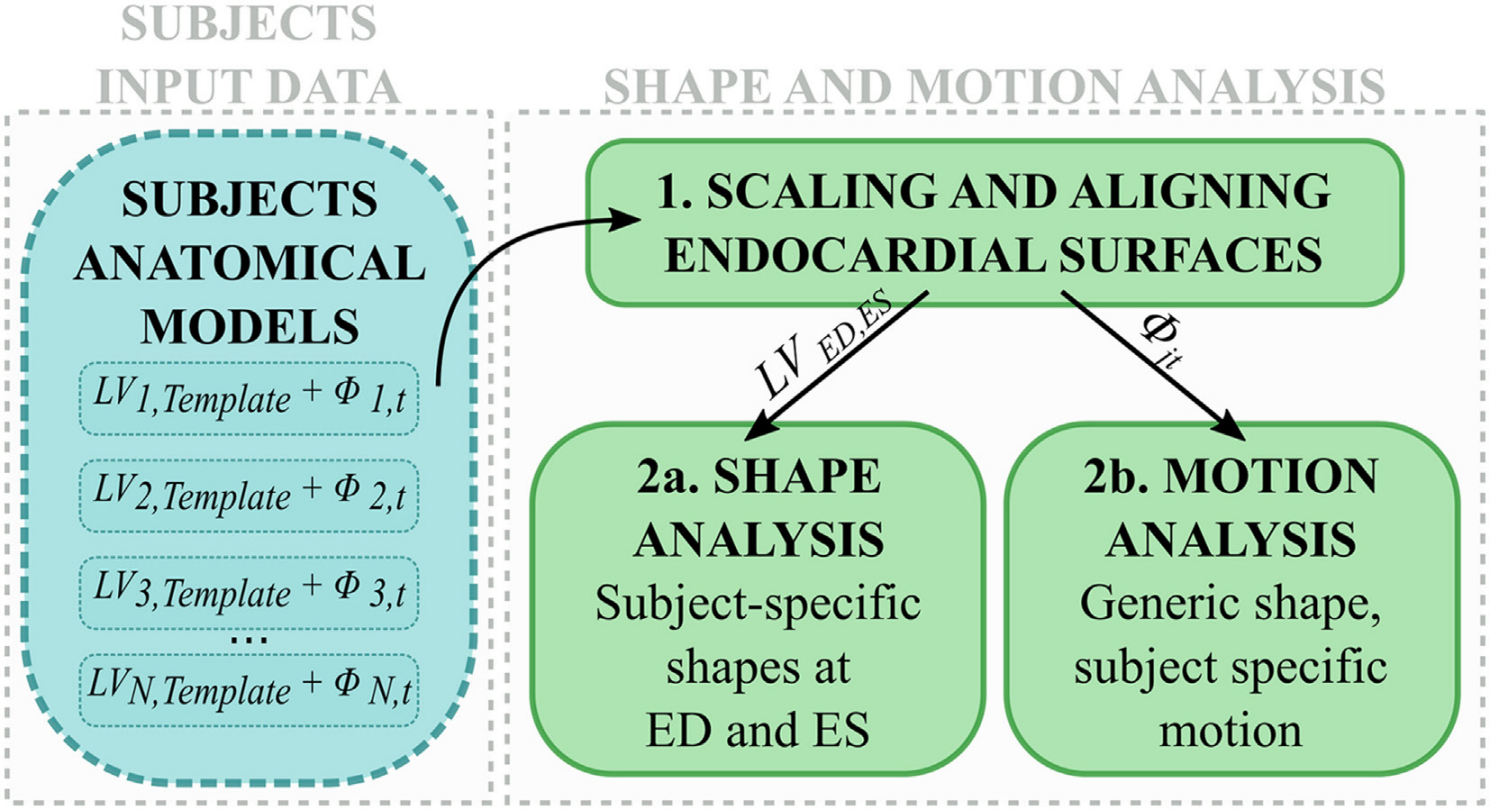
**After scaling and alignment (1), subject anatomical models were used to perform (2a) shape analysis on end-diastolic and end-systolic shapes, and (2b) motion analysis after subject-specific shape was removed**.

The workflow developed in this study is illustrated in Figures 1 and 2. The first step involved processing of the subject image data (Figure 1, central panel). Highly detailed shape features from the WH sequence were combined with the motion from cine images, in order to provide synthetic high-resolution 3D images throughout the full cardiac cycle (i.e., Motion propagation step in Figure 1). An automatic segmentation method previously developed was used to label the main cardiac structures of interest (i.e., Automatic segmentation step in Figure 1), and triangulated meshes were obtained from the segmented LV masks (see 2.3.1). Each subject LV *j* was represented in terms of an anatomical model, i.e., a template mesh *LV_j,Template_* and a set of deformations *ϕ_jt_* warping the template to each temporal occurrence *t* of the cardiac cycle (i.e., Anatomical model step in Figure 1 and paragraph 2.3.2).

In the second step, LV anatomical models generated for each subject were processed to perform shape and motion analysis separately (Figure 2). *LV_j,Template_* from each subject *j* was scaled, rigidly aligned (Figure 2, 1), and inputted into our SSA framework. For shape analysis alone (Figure 2, 2a), ED and ES meshes were analyzed across subjects (see 2.3.3). For motion analysis (Figure 2, 2b), each estimated subject-specific motion was used to deform a newly calculated general template shape (*LV_SuperTemplate_*) from the full population geometrical inputs, allowing study of subject-specific motion without the influence of the subject-specific shape. The resulting temporal datasets were analyzed with an SSA approach similar to that used for pure shape (see 2.3.4).

### 2.3 Computational Framework

#### 2.3.1 Subject Data Processing: Motion Propagation and Automatic Segmentation

An automatic image-processing pipeline able to exploit and combine both 3D WH and temporal information provided by cine CMR images was developed and applied to each subject set of images. First, initial 3D images of the heart (*CINE_jt_*) were obtained from the cine SAX stack via combination of all the images acquired in the same phase of the cardiac cycle, *t*, based on the trigger time. DICOM tags referring to slice spacing and orientation were used to guarantee correct slice alignment. Despite their low resolution in the long axis plane, *CINE_jt_* images retain the temporal information of the LV motion throughout the full cardiac cycle. To integrate the motion information with the detailed 3D spatial resolution provided by the WH dataset *WH_j_*, synthetic WH images (*WH_Syn,jt_*) were obtained for each of the 20 acquisition frames via non-rigid image registration (17), i.e., a transformation in which *WH_j_* is deformed and morphed to replicate the LV configuration represented in each *CINE_jt_*. More specifically (18), all the *CINE_jt_* were scored depending on the resemblance with *WH_j_*, the latter quantified by the sum of squared difference between voxel intensities similarity measure (*SSD*). A threshold was set as *SSD_Th_* = 0.5 * *SSD_Max_*, where *SSD_Max_* was the maximum value found for each subject, and *CINE_jt_* with *SSD_jt_* < *SSD_Th_* were classified as “highly similar,” while the others as “poorly similar.” In case of highly similar *CINE_jt_* (usually in the diastolic phase), *WH_Syn,jt_* was generated by directly registering *WH_j_* to *CINE_jt_*. In case of poorly similar *CINE_jt_* (usually in the systolic phase) *WH_Syn,jt_* was generated by registering the previously obtained *WH_Syn,j_*_(_*_t_*_−1)_ to *CINE_jt_*. Image registration and transformation were performed exploiting the open-source library niftyreg (19). Each *WH_Syn,jt_* was segmented using an in-house atlas-based segmentation method previously validated (20, 21), able to automatically label the main cardiac structures of interest.

#### 2.3.2 Subject Data Processing: Creating LV Subject-Specific Anatomical Models

For each subject, LV myocardium masks obtained from segmentation were converted in surface meshes (*LV_jt_*) and used to build the subject anatomical model using the code Deformetrica (22).

**Figure 3:**
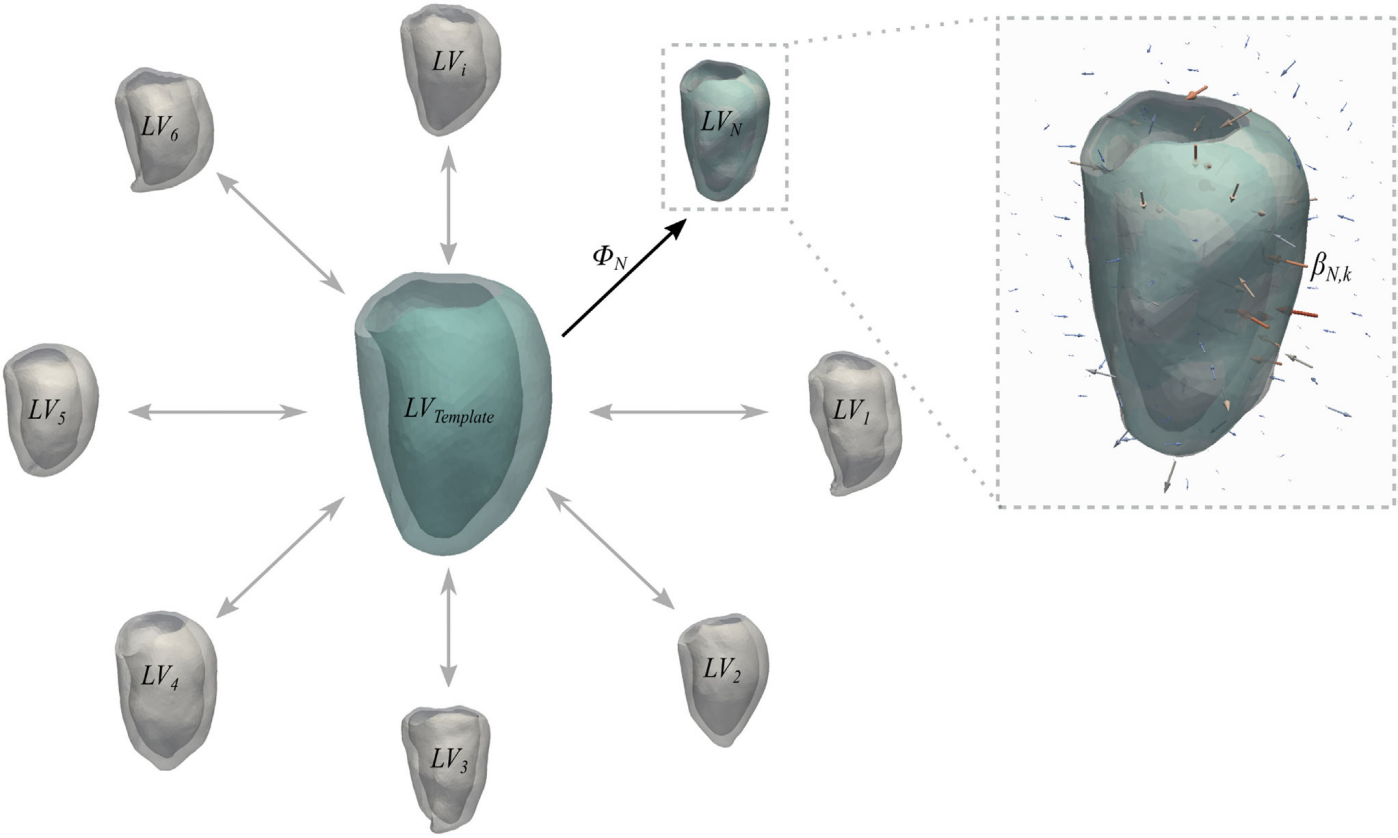
**Schematic representation of the concept of anatomical model**. Template and deformations *ϕ_i_* toward each shape are estimated with an alternate registration algorithm. The template is initialized as the mean shape and the deformations *ϕ_i_* are estimated using a Large Deformation Diffeomorphic Mappings framework (23, 24). Each deformation *ϕ_i_* is defined by a set of momenta vectors *β_ik_* (arrows in the magnified panel) located at the control points grid.

Briefly, a generic anatomical model is the ensemble of a *Template* mesh, which represents the 3D average of the input shapes, and a set of deformations *ϕ_i_* of the 3D space warping the template to each one of the input shapes (Figure 3). Specifically, deformations are represented by a set of vectors—namely, momenta *β_ik_*—attached in the 3D space to a control point grid. For the latter, the amount of control points *k* is chosen by the user, while their position is automatically optimized to densely sample the most variable regions of the template shape (22). Parameters to be set by the user are the resolution *λ_w_* of the shape representation (i.e., how fine are the details we want to capture) and the stiffness of the deformation *λ_v_*, both in millimeters (9).

In this case, the template mesh (*LV_j,Template_*) represented the time-averaged subject-specific ventricle shape, and the momenta warped the template to each temporal occurrence within the subject-specific cardiac cycle. As suggested by Bruse et al. (7), the parameters (i.e., resolution *λ_w_* and transformation stiffness *λ_v_*) required to run the computation of each subjects anatomical model were iteratively tuned to maximize the matching of the template with the original shapes. This was quantified by computing the average euclidean surface distance between computed (i.e., template-matched) and original shapes. These values were further averaged between the 20 frames to give a unique value for each subject. Surface distances were computed as the pointwise minimum distance of the input surface from a reference surface by exploiting The Vascular Modeling Toolkit (25) (VMTK, Orobix, Bergamo, Italy; www.vmtk.org) function vmtksurfacedistance. In order to minimize the effect of size and orientation on the next steps of the analysis, all anatomical models were first scaled with respect to each *LV_j,Template_* endocardial volume, and then rigidly aligned (26) throughout a generalized procrustes analysis (27) iterative process on the *LV_j,Template_* endocardial surfaces, implemented with the functions available in the open-source library niftyreg (19).

#### 2.3.3 Shape Analysis

In order to quantitatively describe anatomical shape and motion variations within a population, we herein extensively adopted an SSA approach (7, 11). As the variation of the data within an anatomical model is described by a large number of momenta vectors, output data are not trivial to analyze and interpret. Therefore, the second step required to analyze variability is to apply dimensionality reduction [i.e., PCA (28)] to the momenta vectors, a common mathematical technique that discards any redundant information while keeping principal contributors to variability. Specifically, momenta vectors are projected onto the space that maximizes their covariance, and only the components—also called modes—that contain most of the information are retained as descriptors. By deforming the template shape along the derived modes toward negative and positive extremes of each mode (±2*σ*), it is possible to visualize and thus qualitatively assess the dominant global and local shape variations characterizing the examined population. The amount of information carried for each subject by each mode is summarized in the shape vector, i.e., a vector where each entry represents how much the template has to be deformed along the corresponding mode in order to match each specific input shape (7, 10). Analysis of the shape vectors allows quantification of the differences in shape within the population.

**Figure 4:**
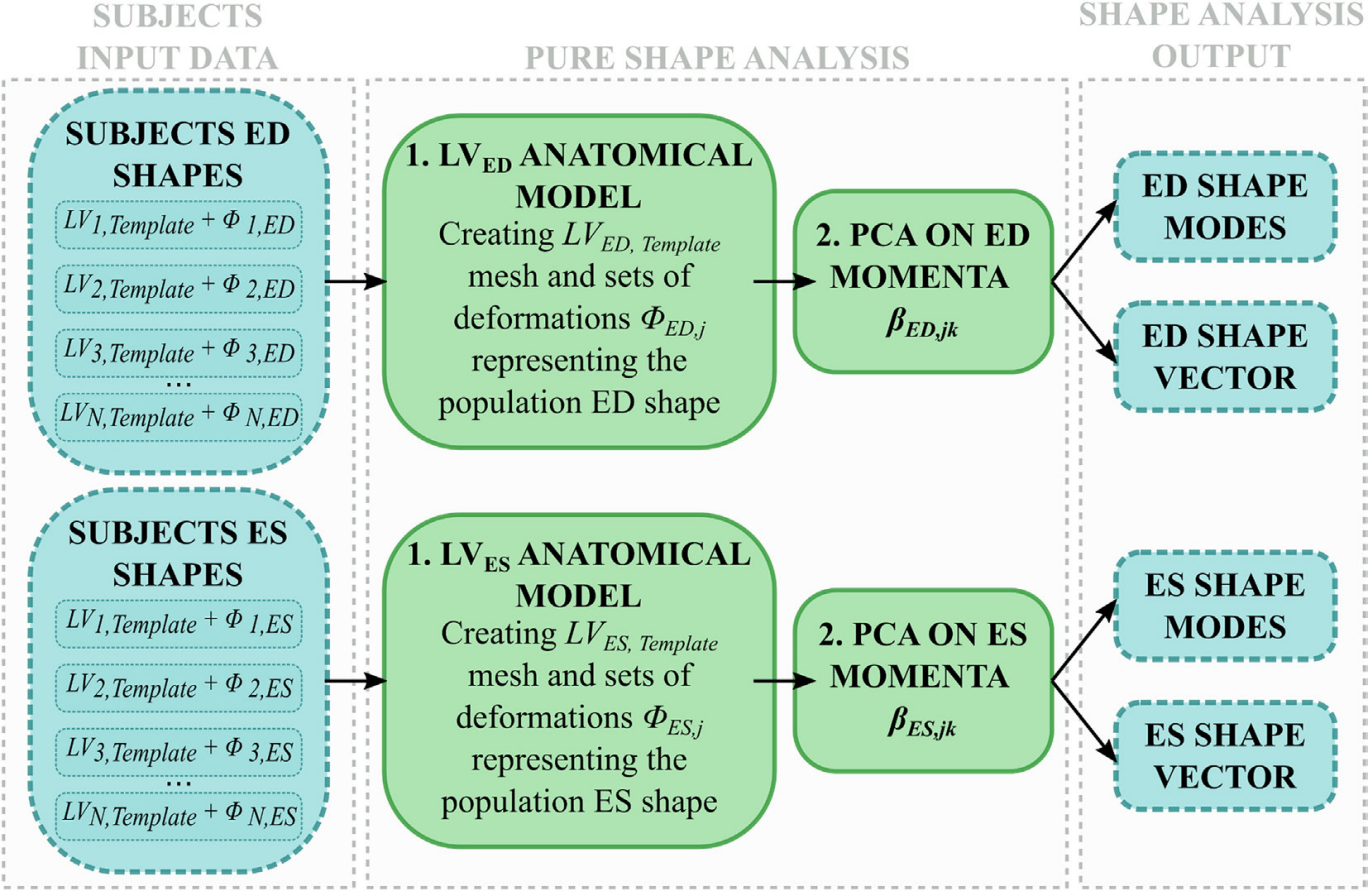
**Shape analysis pipeline: for each subject, only ED and ES shapes are used to (1) build an anatomical model representing the *LV_ED_, LV_ES_* shape variability within the population; (2) the momenta vectors are decomposed with a PCA and the resulting shape modes and shape vector are analyzed**.

Shape analysis was applied separately to the end-diastolic (ED) meshes (*LV_ED_*) and the end-systolic (ES) meshes (*LV_ES_*) (Figure 4). The two groups (i.e., 8 shapes each) were separately processed using the abovementioned SSA framework, i.e., two anatomical models were obtained from only ED and ES shapes, respectively, generating *LV_ED,Template_, LV_ES,Template_*, and associated deformations (*ϕ_ED,j_, ϕ_ES,j_*). Both the anatomical models modes and shape vector were then computed and analyzed.

The shape variability expressed by each mode (*LV_ED,Template_* ± 2*σ_modeED,x_, LV_ES,Template_* ± 2*σ_modeES,x_*) was first qualitatively observed. This visual description, in conjunction with the respective shape vector coefficients, was then used to numerically characterize each subject LV 3D shape at ED or ES, eventually highlighting shape similarities and differences within the joint population of patients and control group.

#### 2.3.4 Motion Analysis

**Figure 5:**
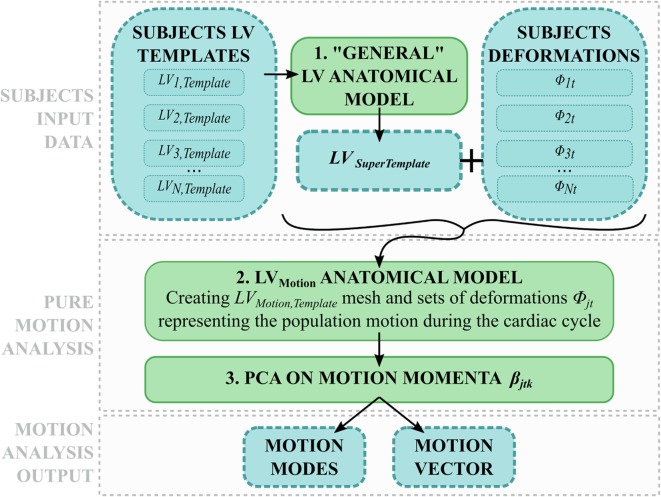
**Motion analysis pipeline: (1) each subject *LV_j,Template_* is used to compute a general LV shape called *LV_SuperTemplate_*. (2) The latter is deformed with each subject-specific deformation, and all the obtained shapes are used to build a motion anatomical model (2). The resulting momenta vectors are decomposed with PCA (3), generating motion modes and motion vector describing each subject motion within the cardiac cycle**.

To extend the SSA framework to the analysis of subject-specific cardiac contraction, motion was interpreted as an ensemble of relatively small, periodical variations of the same shape during the cardiac cycle. In order to describe motion *per se* without geometrical confounding factors, the effect of the subject-specific shape had to be removed from each anatomical model, hence retaining only the subject-specific motion information. Therefore, we exploited the momenta vectors and control point grids computed within each subject anatomical model (see 2.3.2), and we used them to deform a generic template ventricle (namely, *LV_SuperTemplate_*), obtained as the average shape of all the 8 subjects *LV_j,Template_* (Figure 5, 1). As a result, *LV_SuperTemplate_* deformed throughout the cardiac cycle following each subject-specific contraction pattern. The same SSA framework described above (see 2.3.3) was then applied to this set of 160 shapes (20 frames for each of the 8 subjects, Figure 5).

The motion patterns characterized by each mode were first qualitatively assessed by visual observation. By exploiting the quantitative information given by the motion vector, each subject’s LV contraction pathway was then numerically described in terms of the identified motion modes. This allowed us to consistently characterize each subject LV dynamics independent from its shape within the same mathematical framework, ideally providing insight into ventricular function and dysfunction.

## 3 Results

### 3.1 Subject Data Processing

**Figure 6:**
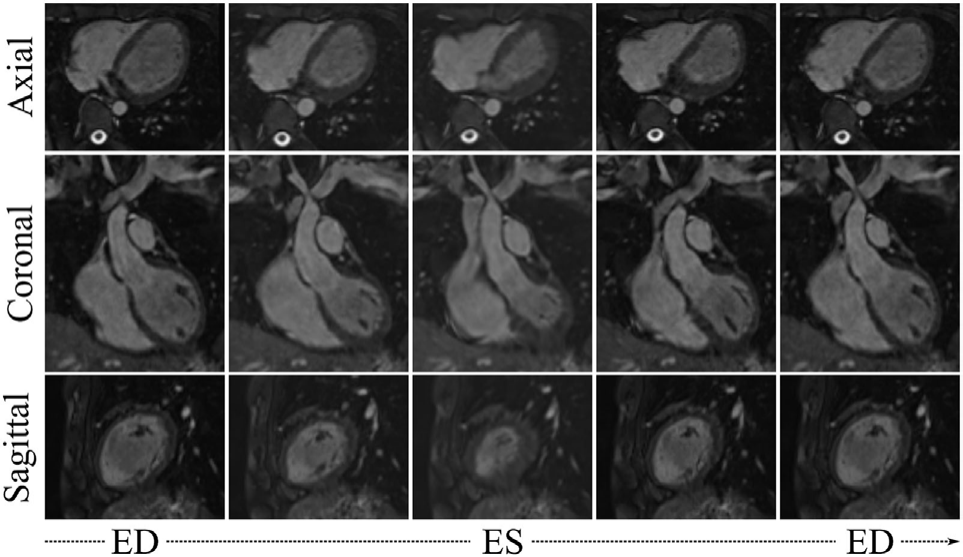
***WH_Syn,jt_* from 5 evenly spaced frames of the cardiac cycle of the subject *j* = *Control*_2_, obtained by propagating the subject’s motion from the cine dataset onto the high-resolution WH image**.

**Figure 7:**
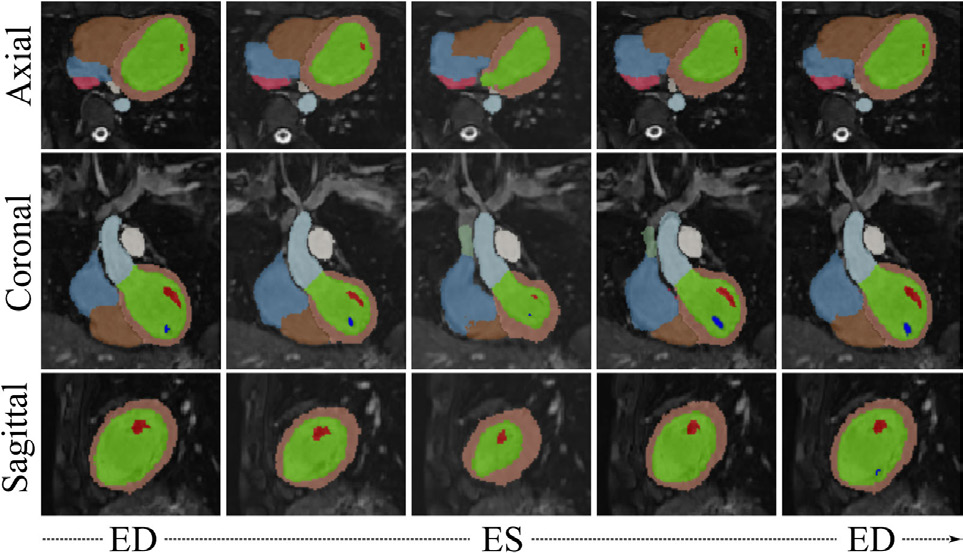
***WH_Syn,jt_* with overlaid automatic segmentation from 5 evenly spaced frames of the cardiac cycle of the subject *j* = *Control*_2_**. Color code for main segmented structures: lemon-green, LV blood pool; orange, LV myocardium; red and dark blue, papillary muscles; light blue, aorta; brown, right ventricle; azure, right atrium; green, superior vena cava; pink, inferior vena cava; white, pulmonary trunk.

Visual results from the image processing pipeline are shown in Figures 6 and 7 for a healthy subject (*Control*_2_), where we can observe realistic systolic contraction and myocardial wall thickening on synthetic images (Figure 6), as well as an accurate identification of the main cardiac structures performed by our automatic segmentation method (Figure 7). Considering our main focus on shape and shape variations more than on cardiac volume quantification, our method allows fast and consistent processing and segmentation of a large amount of WH data, which would be otherwise challenging to process manually.

**Figure 8:**
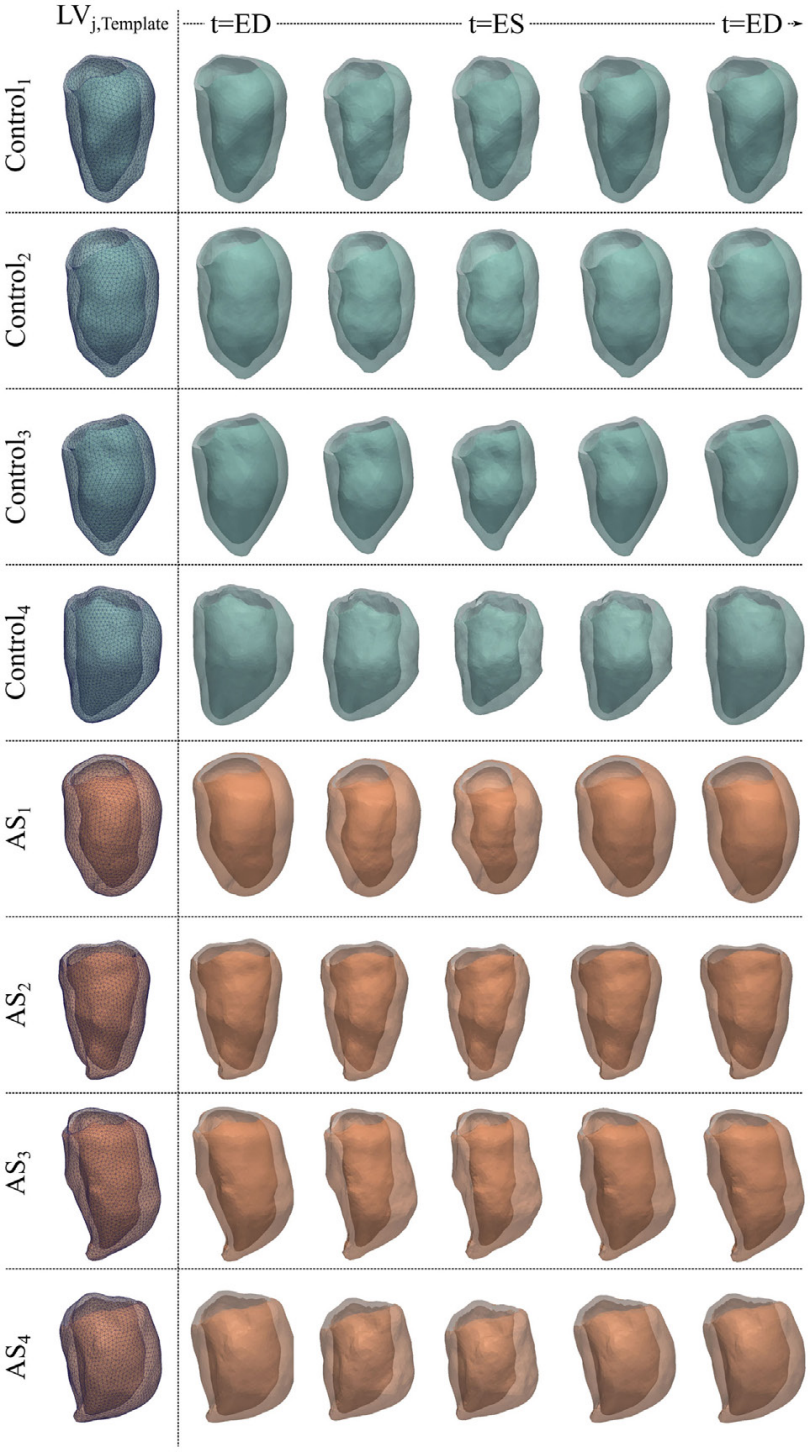
***LV_jt_* meshes for the 4 control and 4 AS subjects obtained by deforming subject-specific *LV_j,Template_* (first column) with the deformations estimated during the anatomical model computation, to reproduce 5 evenly spaced frames of the cardiac cycle illustrated in Figures 6 and 7 for each subject**.

The parameters required to run the computation of each subject anatomical model were set to *λ_w_* = 13 mm and *λ_v_* = 23 mm, leading to an intersubject averaged surface distance between computed (i.e., template-matched) and original shapes of 1.0 mm (*max surface distance* = 2.5 mm in *AS*_1_, *min surface distance* = 0.4 mm in *AS*_2_, *standard deviation* = 0.8 mm). The results of anatomical model computation are shown in Figure 8 for each subject. In particular, we show the *LV_j,Template_* shape and examples of meshes resulting from morphing the template with 5 of the estimated deformations, evenly spaced across the cardiac cycle.

### 3.2 Shape Analysis

#### 3.2.1 ED Shape Analysis

**Figure 9:**
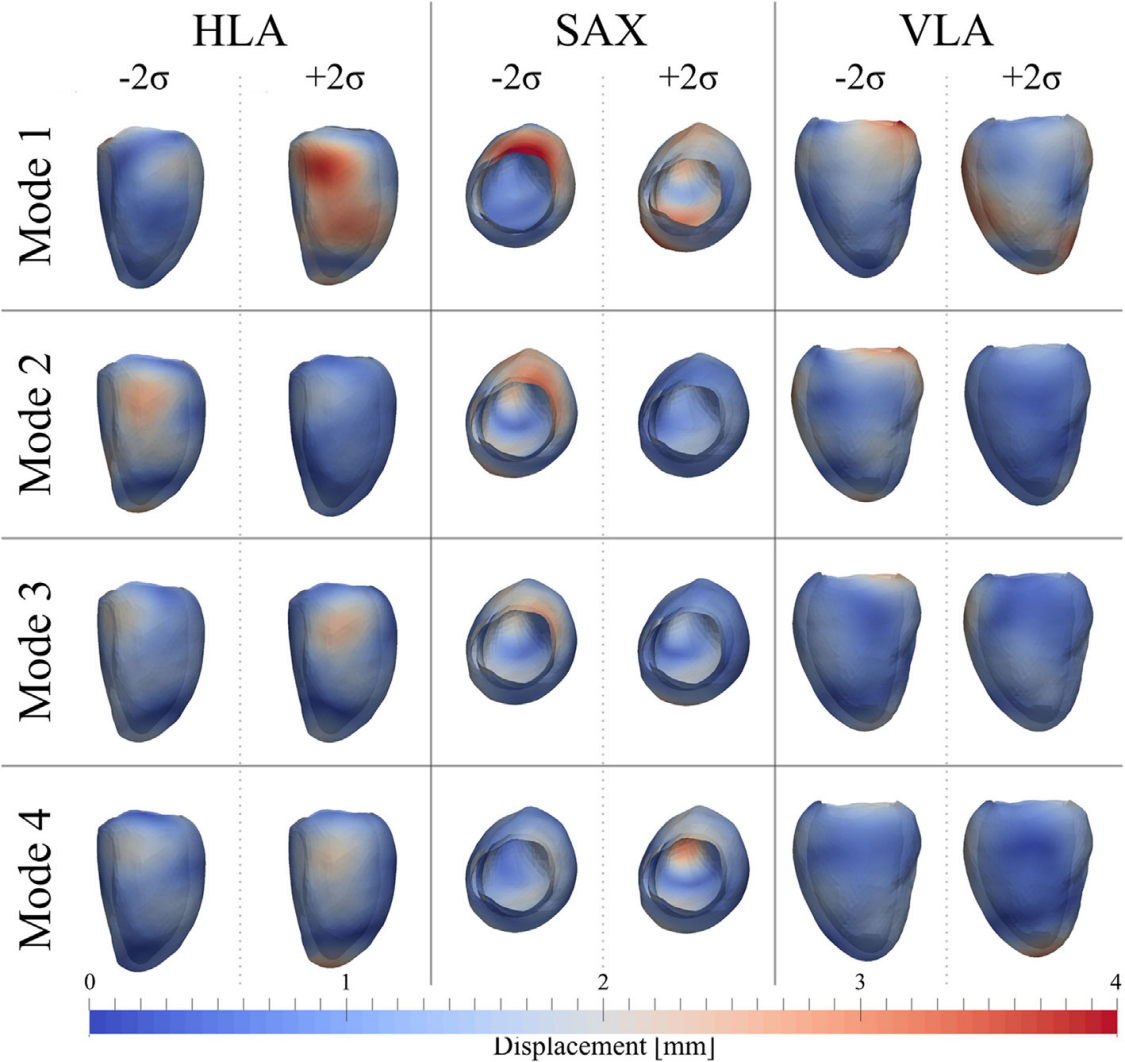
**Extreme features (±2*σ*) of each of the first 4 modes of the shape analysis on LV shapes at ED are shown in the canonical cardiac views (horizontal long axis (HLA), SAX, and vertical long axis (VLA))**. Each depicted shape was obtained by morphing the template shape with the extreme deformation represented by each mode. Colormap represents the distribution of regional deformations within each mode, obtained by deforming the ED template shape along the mode (red, high deformation; blue, low deformation).

After PCA, the first four modes were considered the most relevant for ED shape analysis, accounting for 76% (28%*Mode*_1_, 19%*Mode*_2_, 16%*Mode*_3_, 13%*Mode*_4_) of the total shape information present in the population. Figure 9 illustrates the extreme features (±2*σ*) represented by each mode, in the standard cardiac views. Each depicted shape was obtained by morphing the template shape with the extreme deformation represented by each mode, and the colormap identifies the regions where the highest deformation (in red) occurs. Upon visual inspection, all modes showed a general rounding and circumferential expansion of the basal-mid walls, while the apical region was thinning in *Mode*_1−2_*_σ_* and *Mode*_4−2_*_σ_*, and rounding in all the other modes, causing a shortening of the ventricle. *Mode*_1_, which reflects the most dominant shape variation in the cohort of patients/controls, was locally characterized by a bulging of the basal inferior and inferolateral walls and the dilation of the mitral valve annulus in *Mode*_1 − 2_*_σ_*, and by a local outward expansion of the mid anteroseptum and anterior wall in *Mode*_1 + 2_*_σ_*. *Mode*_2 − 2_*_σ_* had an outward dilation and bulging of the mid anteroseptum, the basal inferior and inferolateral walls, while *Mode*_2 + 2_*_σ_* did not show substantial local deformations. In *Mode*_3 − 2_*_σ_*, there was a bulging of the basal inferior wall, while in *Mode*_3 + 2_*_σ_*, we observed a mild expansion of the mid anterior wall. Finally, the region of the anteroseptum was predominantly bulging in *Mode*_4 − 2_*_σ_*, while the mid anterior wall was expanding outward in *Mode*_4 + 2_*_σ_*.

**Figure 10:**
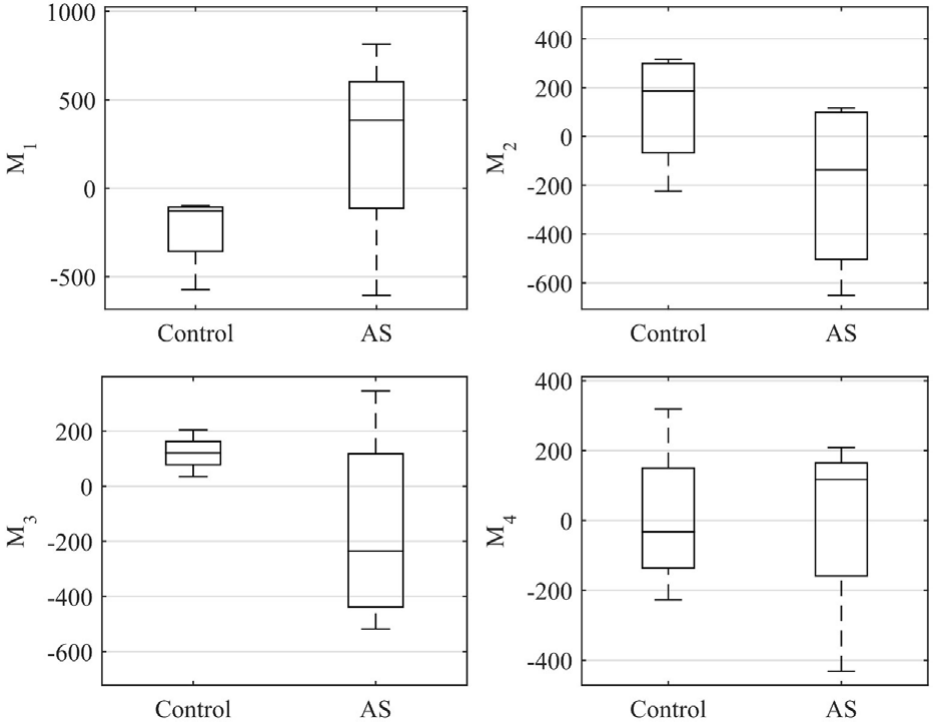
**Boxplot showing the variability of the coefficients of the shape vector of *LV_ED_* shape analysis for the first 4 modes**. Single values are reported in Table 2. On each box, the central mark is the median, the edges of the box are the 25th and 75th percentiles, the whiskers extend to the most extreme data points not considered outliers.

Figure 10 shows a boxplot representing the variability of the shape vector entries (Table 2) for control group and AS patients, allowing to describe each subject LV shape numerically according to the modes previously illustrated. In this example population, overall the control group had an ED shape characterized by the negative extreme of *Mode*_1_, the positive extreme of *Mode*_2_ and *Mode*_3_, and a predominantly negative extreme of *Mode*_4_. As expected, the AS patients showed more variability within each mode, with some subjects sitting within the control range of variability, but single subjects being clearly outside the control range. Given the small sample size (n = 4 for each population), no significant difference (*p* > 0.05 from Mann–Whitney U test) could be found between the two groups medians, for each of the mode. Following the visual results, ED shape of the AS patients was therefore characterized by a local outward expansion of the mid anteroseptum and anterior wall (*Mode*_1 + 2_*_σ_*), outward dilation and bulging of the mid anteroseptum, the basal inferior and inferolateral walls (*Mode*_2 − 2_*_σ_* and *Mode*_3 − 2_*_σ_*) and mid anterior wall (*Mode*_4 + 2_*_σ_*), according to the descriptions above.

**Table 2:** **Coefficients of the shape vector of *LV_E__D_* shape analysis for the first 4 modes**.

Subject	Mode_1_	Mode_2_	Mode_3_	Mode_4_	Subject	Mode_1_	Mode_2_	Mode_3_	Mode_4_
Control_1_	−140	316	121	−20	AS_1_	816	117	−359	209
Control_2_	−98	282	204	319	AS_2_	−607	−356	−518	120
Control_3_	−574	91	121	−46	AS_3_	390	81	−111	−432
Control_4_	−116	−224	34	−227	AS_4_	380	−652	346	114
Median	−128	186	121	−33	Median	385	−137	−235	117
IQR	137	279	43	156	IQR	363	520	402	164

*Control subjects (left) and AS (right). Within each mode, data are summarized in median ± IQR*.

#### 3.2.2 ES Shape Analysis

**Figure 11:**
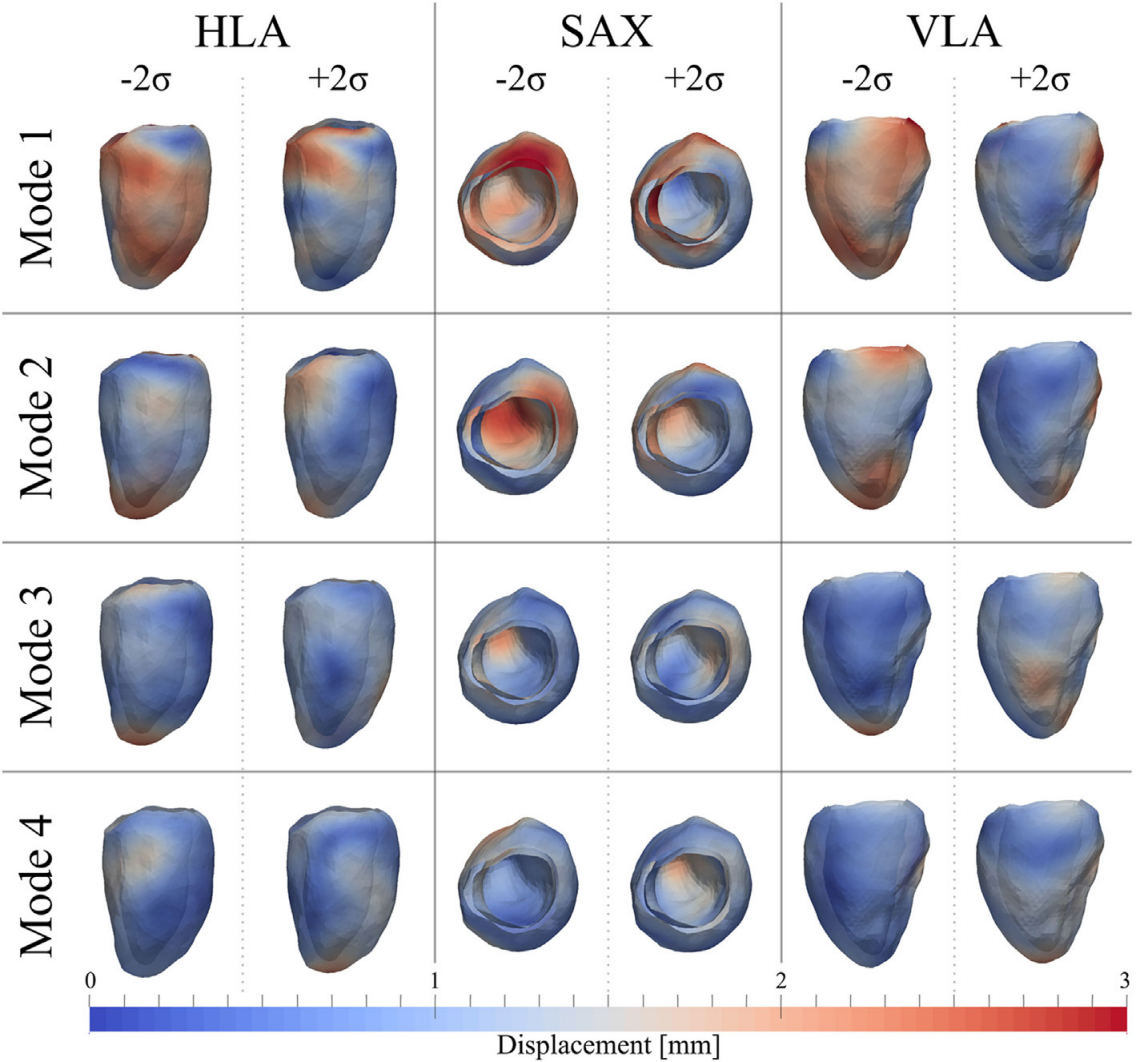
**Extreme features (±2*σ*) of each of the first 4 modes of the shape analysis on LV shapes at ES are shown in the canonical cardiac views (HLA, SAX, and VLA)**. Each depicted shape was obtained by morphing the template shape with the extreme deformation represented by each mode. Colormap represents the distribution of regional deformations within each mode, obtained by deforming the ES template shape along the mode (red, high deformation; blue, low deformation).

As for the ED shape analysis, the first four modes were considered the most relevant for ES shape analysis, capturing 75% (28%*Mode*_1_, 17%*Mode*_2_, 16%*Mode*_3_, 14%*Mode*_4_) of the shape variability in our population. The deformations represented by each mode are shown in Figure 11, as for the ED case. In ES, shape of *Mode*_1 − 2_*_σ_* was characterized by an overall smaller size, especially locally at the basal inferior and inferolateral walls. In *Mode*_1 + 2_*_σ_*, the base was shortening and the mid walls bulging outward, while the mitral valve annulus was circumferentially smaller, and the basal infero- and anteroseptum and mid inferolateral walls were reduced. In *Mode*_2 − 2_*_σ_*, the apex and the apical walls were bent more anteriorly and toward the right, the basal lateral wall was positioned more posteriorly, and the mitral valve annulus was more dilated, while in *Mode*_2 + 2_*_σ_*, the septum was positioned more posteriorly. Shape in *Mode*_3 − 2_*_σ_* was generally shorter, the apex rounder and slightly bent anteriorly and toward the right, while in *Mode*_3 + 2_*_σ_*, the mid walls were circumferentially shrunk, especially in the apical lateral wall. Finally, in *Mode*_4 − 2_*_σ_*, the septal wall was slightly reduced, especially in the mid inferoseptum, and in *Mode*_4+2_*_σ_*, the apex was rounder and bent more posteriorly.

**Figure 12:**
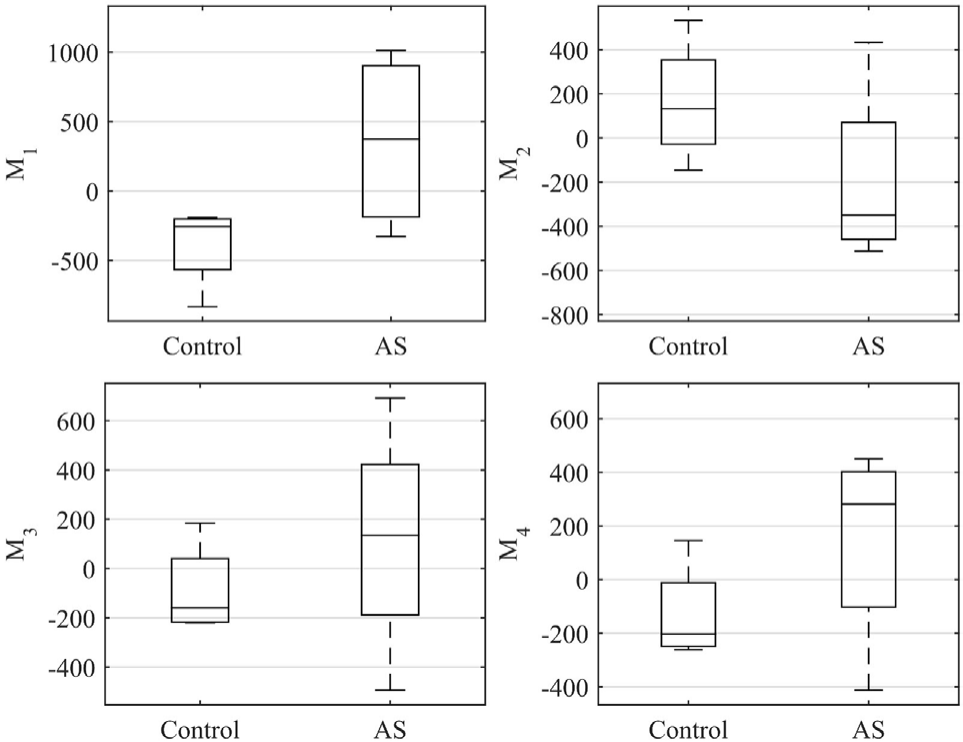
**Boxplot showing the variability of the coefficients of the shape vector of *LV_ES_* shape analysis for the first 4 modes**. Single values are reported in Table 3. On each box, the central mark is the median, the edges of the box are the 25th and 75th percentiles, the whiskers extend to the most extreme data points not considered outliers.

The ES shape vector entries variability for control group and AS patients are represented in the boxplot in Figure 12 and Table 3. As for the ED case, variance of the control group coefficients was overall less than that for the AS patients. In particular, coefficients of the control group were all in the negative extreme for *Mode*_1_, predominantly positive in *Mode*_2_ and predominantly negative in *Mode*_3_ and *Mode*_4_. Even though shape vector entries for single AS patients showed differences from the control group, not all the shape modes could clearly distinguish the two groups, similar to the ED shape analysis. Also in this case, the small sample size (n = 4 for each population) did not allow to show significant difference (p > 0.05 from Mann–Whitney U test) between the two groups medians, for each of the mode.

**Table 3:** **Coefficients of the shape vector of *LV_E__S_* shape analysis for the first 4 modes**.

Subject	Mode_1_	Mode_2_	Mode_3_	Mode_4_	Subject	Mode_1_	Mode_2_	Mode_3_	Mode_4_
Control_1_	−190	175	−220	−170	AS_1_	1,012	434	117	208
Control_2_	−299	533	−104	146	AS_2_	−328	−292	693	355
Control_3_	−834	90	184	−262	AS_3_	792	−406	153	−413
Control_4_	−212	−145	−214	−237	AS_4_	−45	−512	−494	450
Median	−256	133	−159	−203	Median	374	−349	135	281
IQR	226	234	184	152	IQR	963	322	323	326

*Control subjects (left) and AS (right). Within each mode, data are summarized in median ± IQR*.

### 3.3 Motion Analysis

**Figure 13:**
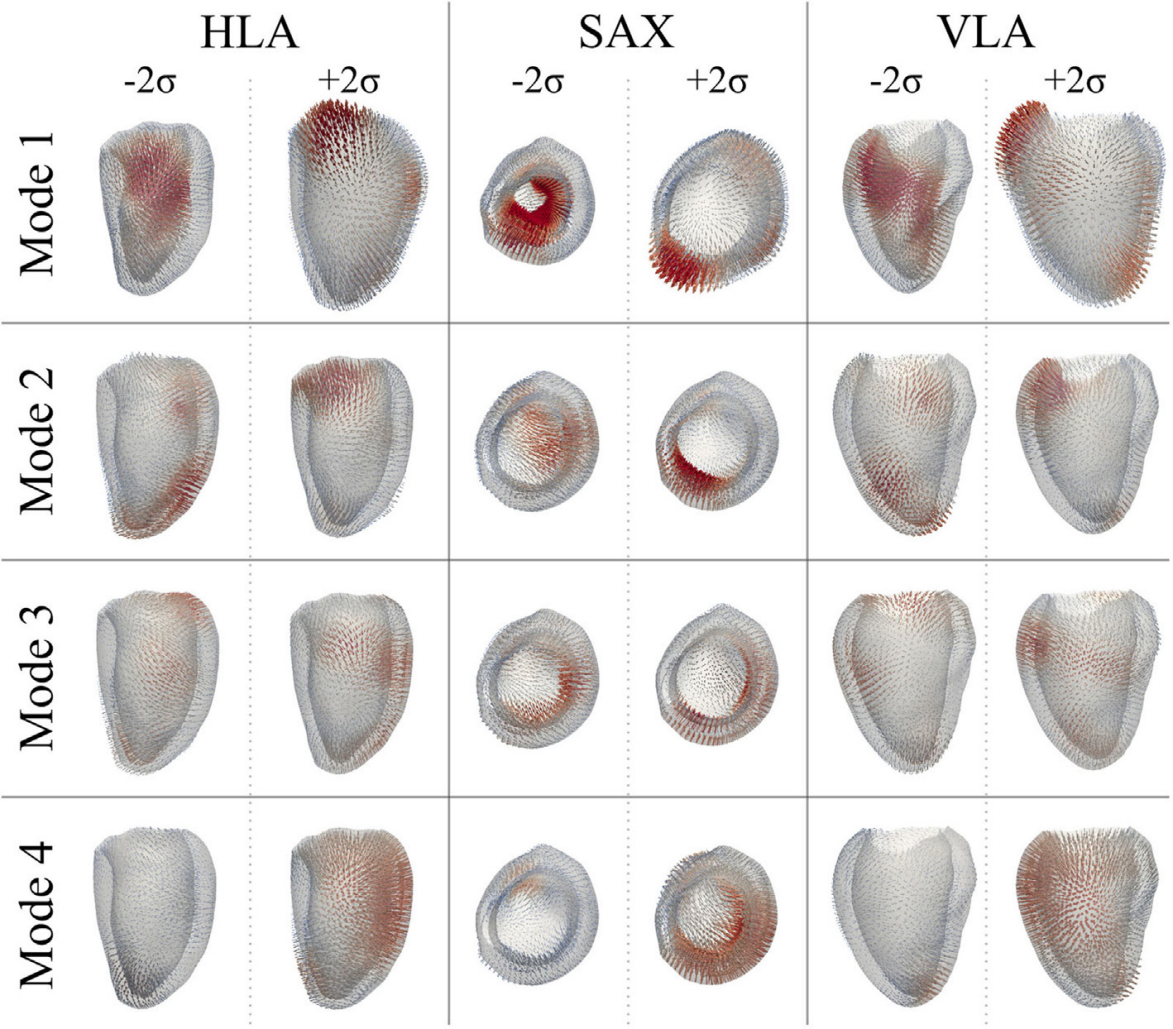
**Extreme motion patterns (±2*σ*) represented by each mode of the motion analysis on LV shapes are shown in the canonical cardiac views (HLA, SAX, and VLA)**. Each depicted shape was obtained by morphing the *LV_SuperTemplate_* shape with the extreme deformation represented by each mode. Arrows represents the direction and magnitude of the movement from the template (red color highlights the regions of highest motion).

After PCA, the first four modes were considered the most relevant for motion analysis alone, accounting for 70% (52%*Mode*_1_, 7%*Mode*_2_, 6%*Mode*_3_, 5%*Mode*_4_) of the population total variability in motion information. Figure 13 illustrates the extreme motion patterns represented by each mode, in standard cardiac views. Similar to the shape example, here each figure was obtained by morphing *LV_SuperTemplate_* with the extreme deformations represented by each mode (±2*σ*), with the arrows identifying local motion direction and magnitude (red color highlights the regions of highest motion). After qualitative visual assessment, *Mode*_1_ represented the overall ventricular contraction (*Mode*_1 − 2_*_σ_*) and expansion (*Mode*_1 + 2_*_σ_*), which characterize the systole–diastole alternation typical of the cardiac cycle. On top of this general trend, contraction in *Mode*_1 − 2_*_σ_* prevailed in regions such as the basal and mid anterior wall, but also mid lateral and mid inferior walls, while the basal anteroseptum was the most affected by expansion in *Mode*_1 + 2_*_σ_*. Toward the negative extreme, in *Mode*_2 − 2_*_σ_* the apex and apical portion of the lateral wall moved rightward, and the basal anterolateral wall moved up, anteriorly and rightward. Toward the positive extreme (*Mode*_2 + 2_*_σ_*), the basal anteroseptum and anterior wall moved downward, while the apical septum moved posteriorly. *Mode*_3 − 2_*_σ_* was characterized by the basal portion of the lateral wall moving upward and anteriorly, the mid anterior and anterolateral walls moving upward and rightward, and the whole apical region moving anteriorly. *Mode*_3 + 2_*_σ_* was described by a downward movement of both the mid anterior wall and the basal lateral wall, and by an upward-rightward movement of the apical lateral wall. Finally, in *Mode*_4 − 2_*_σ_*, the apical septum and lateral walls were moving upward and posteriorly, while in *Mode*_4 + 2_*_σ_*, the basal and mid anterior and lateral walls were contracting, with the apical septum moving anteriorly and rightward. Looking at the valves plane, this moved upward in *Mode*_1 + 2_*_σ_, Mode*_2 − 2_*_σ_*, and *Mode*_4 − 2_*_σ_*, while it moved downward in all the other mode occurrences.

**Figure 14:**
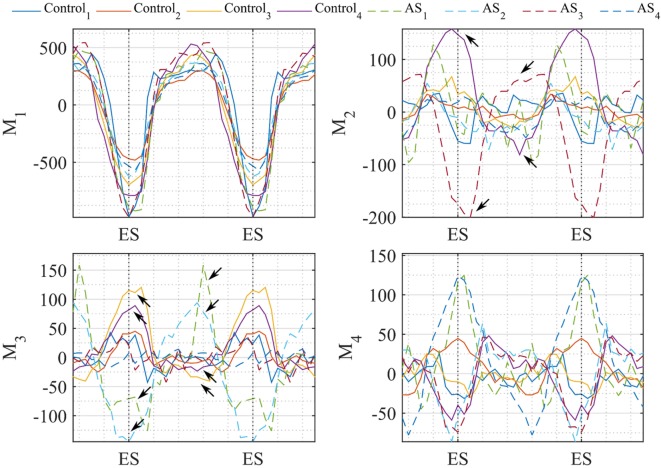
**Coefficients of the motion vector plotted against time (2 cardiac cycles are herein represented)**. Each line represents one subject, i.e., Control subjects are plotted in continuous line and AS in dashed line. Temporal variation of the coefficients resulting from our motion analysis is plotted for the first 4 modes. Arrows highlight the values where coefficients differ mostly between control-AS subjects comparison.

In Figure 14, we plotted the values of the motion vectors over two cardiac cycles to understand which motion pattern was predominant in every subject, and in which part of the cardiac cycle. As a proof of concept, we show the potentiality of this method in quantifying local 3D variations in motion patterns, by showing extreme results obtained with the examined population. The trend of the shape vector coefficients relating to *Mode*_1_ showed to be consistent between all subjects, suggesting that the normal overall pattern of contraction and expansion between systole and diastole is maintained in both patients and control group. *Mode*_2_, mostly accounting for basal and apical motion, resulted to be expressed in a completely opposite way for *Control*_4_ (purple line) and *AS*_3_ (red dashed line), during the whole cardiac cycle. This can be seen in Videos S1–S3 in Supplementary Material, where during the systolic phase the apex of *AS*_3_ strongly moves to the right, and the basal septum of *Control*_4_ moves downward. Extreme behaviors in *Mode*_3_ were expressed by *Control*_3_ (yellow line) and *AS*_2_ (azure dashed line), and in *Mode*_4_ by *AS*_2_, *AS*_3_ and *AS*_1_, *AS*_4_. However, considering the less importance of these modes compared to the first two, the visual effect on the overall motion was more difficult to identify, which is why a quantitative analysis like that shown in Figure 14 could help highlight small issues in LV motion, not easily detectable at visual observation.

## 4 Discussion

In this study, we presented an automated medical image processing pipeline for qualitative and quantitative analysis of LV 3D shape and motion patterns from clinically acquired CMR data, and apply it to a sample population of AS patients and aged-matched healthy volunteers. The main novelty of this work is the development of an image analysis framework that allows combination of temporal (cine) and high resolution spatial information (WH) for 3D subject-specific shape and motion analyses, which are usually considered separately due to the lack of comprehensive and high-resolution 4D CMR image sequences. Dominant shape and motion patterns in both diseased and healthy cohorts were visualized in 3D and could be quantified for in-depth assessment of cardiac function. Results showed that in congenital repaired aortic stenosis, pure analysis of cardiac morphology can be complemented by detailed motion analysis, highlighting regional differences in ventricular contraction patterns.

Since our approach is based on image sequences currently acquired in clinical practice, it does not require further acquisition protocols or increased scanning time. Our pipeline allows using the full image data provided by clinical CMR acquisition, and thus provides more insight than each image set by itself. Shape analysis has been shown in the literature to be a promising technique for differentiating healthy from pathological subjects, and for quantitatively classifying and describing anatomical shapes (9–12, 29, 30). When applied to ED and ES ventricles in our example population, shape analysis allowed us to observe and quantify differences between AS and controls for single cases. However, due to the small sample size, we were not able to claim any statistically significant difference between the two populations. Specifically, due to the fact that the overall anatomical shape of this set of AS was not excessively abnormal, we do not expect any significant shape difference between the two populations, even in larger cohorts. Generalizing from this result we can hypothesize that, depending on the examined population, shape analysis alone may be not sufficient to robustly classify and describe pathological states based only on anatomy differences, especially in those subjects where the cardiac pathology mostly arises due to contraction/functional deficiency and not as a consequence of abnormal morphology. In this sense, our proposed motion analysis method may represent a new tool to summarize, complement, and enrich the information provided by separate CMR imaging sequences. By removing the subject-specific shape information, retaining only the 3D details related to the subject-specific dynamics applied to a generic representative shape of the population (*LV_SuperTemplate_*), our motion analysis method could become useful for the visualization of differences in motion between healthy and pathological subjects, which may otherwise be hidden behind individual shape features. This tool could hence be used for intuitive, easily comprehensible visualization of motion differences and dominant patterns, even in clinical practice. Moreover, if used in comparison with a quantitative analysis of the motion vector, this technique could numerically highlight cardiac motion differences that may be difficult to catch by eye, hence facilitating objective patient diagnosis.

The main limitation of this work is the small sample size used to test the computational framework. With a larger number of healthy subjects, it would be possible to build a comprehensive healthy population atlas, which would allow further, more elaborate statistical analysis. We are currently working on increasing the number of subjects to be included in the tested population, and also on performing further validation. It is to be noted that our analysis pipeline is independent from a specific anatomical region of the heart, i.e., it can be used for shape and motion analysis on structures different from the LV, provided that these are imaged in both WH and cine CMR sequences. Other than for better understanding the pathology, results (i.e., in particular motion propagation plus PCA) could also be used as input for structural or computational fluid dynamics computer simulations with moving boundaries, in particular to provide subject-specific geometries and boundary conditions for realistic analysis of ventricular mechanics or interaction with cardiac devices.

In conclusion, this preliminary work demonstrates the feasibility of using statistical shape analysis in combination with motion analysis based on CMR image data and its potential to detect new shape and motion biomarkers, for a detailed visual and quantitative analysis of cardiac function. Our processing pipeline is fully automatic and requires only basic user input, which makes it an attractive alternative to tedious manual segmentation, measurements, and motion mapping as currently done in clinical practice. Applying state-of-the-art automatic segmentation algorithms in conjunction with statistical shape modeling tools to cardiac image data allows detailed analysis of cardiac shape and motion patterns, which may ultimately facilitate and improve diagnosis and understanding of complex cardiac disease.

## Author Contributions

BB designed the study, implemented and tested the computational framework, and drafted the manuscript. JLB supported the implementation of the statistical shape analysis and revised the manuscript. MAZ supported the implementation of the image processing pipeline and revised the manuscript. HNN enrolled the patients and acquired the images used for this study. AMT and SS conceived the study, participated in its design and coordination, and helped to draft the manuscript. All the authors read and approved the final manuscript.

## Conflict of Interest Statement

The authors declare that the research was conducted in the absence of any commercial or financial relationships that could be construed as a potential conflict of interest.

## Supplementary Material

Video S1 **Arrow representation of Mode_2_ throughout the cardiac cycle for Control_4_ (light blue) and AS_3_ (orange) in the HLA view**. One can notice that during the systolic phase the apex of AS_3_ moves to the right, while the basal septum of Control_4_ moves downwards.

Video S2 **Arrow representation of Mode_2_ throughout the cardiac cycle for Control_4_ (light blue) and AS_3_ (orange) in the VLA view**.

Video S3 **Arrow representation of Mode_2_ throughout the cardiac cycle for Control_4_ (light blue) and AS_3_ (orange) in the SA view**.
